# A Rare Clinical Case of Oral White Sponge Nevus and the Associated Challenges in Its Differential Diagnosis

**DOI:** 10.1155/2024/2251450

**Published:** 2024-03-18

**Authors:** E. Deliverska, Marusya Genadieva, B. Yordanov, J. Kirilova

**Affiliations:** ^1^Department of Dental, Oral and Maxillofacial Surgery, Faculty of Dental Medicine, Medical University-Sofia, Sofia, Bulgaria; ^2^Department of Pathology, Medical University-Sofia, Sofia, Bulgaria; ^3^Department of Prosthetic Dental Medicine, Faculty of Dental Medicine, Medical University-Sofia, Sofia, Bulgaria; ^4^Department of Conservative Dentistry, Faculty of Dental Medicine, Medical University-Sofia, Sofia, Bulgaria

## Abstract

White sponge nevus is a rare genetic autosomal dominant disorder characterized by irregular patches of thick, white diffuse plaques, which mainly affects the oral mucosa and, very rarely, the skin or mucosa of the nose, esophagus, and anogenital region. We present a case of oral white sponge nevus in a 62-year-old woman, the differential diagnosis of which was of particular interest due to other similar white oral lesions, some of which are potentially malignant. The lesions were verified histologically. However, no treatment was performed due to the benign and asymptomatic nature of the lesions.

## 1. Introduction

White sponge nevus (WSN) is a rare hereditary mucosal disease and a genodermatosis characterized by asymptomatic white plaques that affect the oral mucosa and, less commonly, the nasal, esophageal, rectal, and genital mucosa. Oral white sponge nevus usually appears early in life before age 20 and affects both sexes equally and affects 1 : 200 000 individuals worldwide [1–4]. WSN, called Cannon's disease, manifests as symmetrically located white or gray diffuse plaques that are thick, with multiple furrows and various textures. These plaques are most often located bilaterally on the buccal mucosa, followed by labial, gingival, and mucous membranes and the floor of the mouth [1–3].

The first case of WSN was reported and described in 1909 by Hyde and Cannon, who later coined the name and published a detailed report on the disease in 1935 [3]. WSN is a very rare, autosomal dominant hereditary disorder as genetic transmission involves mutations in the cytokeratin four and cytokeratin 13 genes that cause typical mucosal keratinization defects. These defects lead to keratin instability and the appearance of hyperkeratotic lesions on nonkeratinized epithelial surfaces [5–7].

Distinguishing white sponge nevus from other malignant, reactive, familial, and congenital diseases is essential. Therefore, the diagnosis of white sponge nevus is often based solely on the clear clinical presentation and subsequent biopsy of mucosal lesions. Because the oral manifestations of the disease can clinically resemble other lesions, a biopsy is a mandatory element of diagnosis [8, 9]. White lesions can be congenital or acquired. Acquired lesions can be local to the oral cavity or a manifestation of a systemic disease; i.e., compared to other lesions, they can have a different etiology, pathogenesis, and prognosis. White lesions can be caused by various diseases and conditions, from benign reactive lesions to more serious dysplastic and malignant neoplasms. Without additional investigations (e.g., microbiological, histological, and immunological), distinguishing between these lesions is difficult due to their similar clinical appearances within the oral cavity. There is no specific treatment for the disorder, and regular follow-up for any changes is important.

An accurate diagnosis is important for preventing delays in treating patients with more serious potentially malignant or malignant lesions.

By presenting this clinical case, this article is aimed at adding to the literature on white sponge nevus. However, rare findings should also be considered in the differential diagnosis of white oral mucosa lesions.

## 2. Case Report

**Figure 1:**
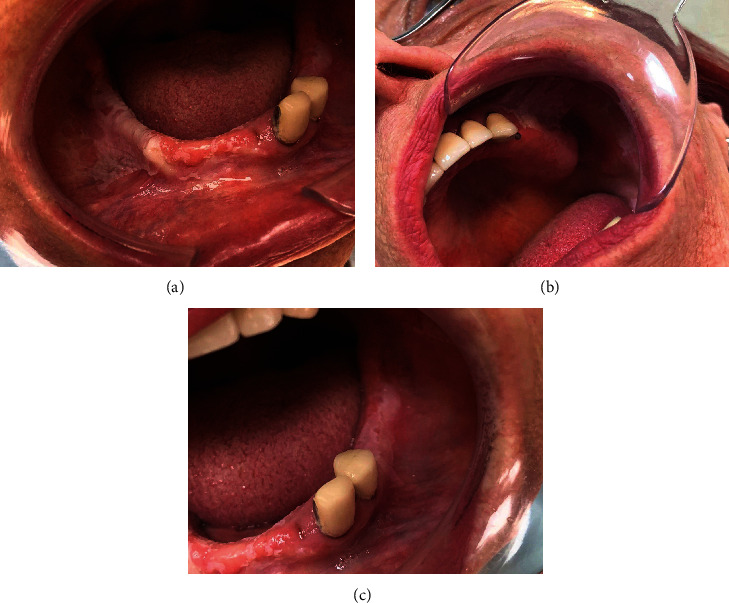
Intraoral photographs of the patient showing the widespread, corrugated, and slightly stippled white lesions with a spongy texture on the (a) right buccal mucosa, (b) upper and lower gingiva, and (c) left buccal mucosa.

**Figure 2:**
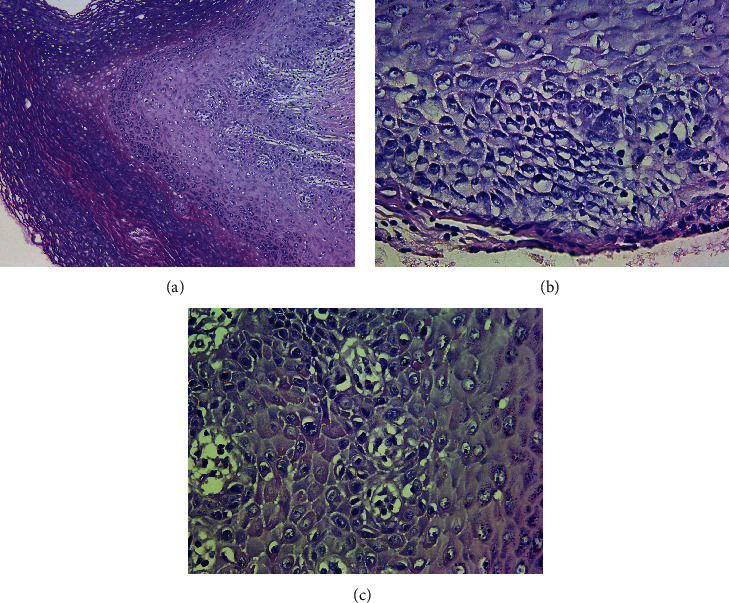
(a) The acanthosis and parakeratosis of the squamous epithelium (hematoxylin and eosin stain, ×10); (b) the extensive vacuolization of suprabasal keratinocytes (hematoxylin and eosin stain, ×40); (c) keratinocytes with dense perinuclear eosinophilic condensation due to the aggregation of intermediate filaments (hematoxylin and eosin stain, ×40).

A 62-year-old woman was referred for consultation as she presented with asymptomatic chronic white lesions of the oral mucosa. The patient signed an informed consent for investigation. Various suggestions for diagnoses (hyperkeratosis, candidiasis, leukoedema, etc.) were made through years without taking material for histological examination. Different topical agents (chlorhexidine gel, Parodium gel, Gengigel, and Daktarin gel) were prescribed without any success. No other family members were affected by similar lesions. The patient had partial upper and lower dentures for years. The lesions were asymptomatic, except for some episodic mild discomfort when eating acidic or spicy food. A clinical examination showed irregular white plaques with well-defined borders and multiple localizations on the buccal mucosa, gingiva, and mucobuccal folds of the upper and lower jaws, with different densities in places (Figure 1). There was no erythema around the plaques, and scraping could not remove them. No other similar lesions were found on other mucous membranes. There was no evidence of any systemic diseases. A histological examination of the lesions revealed squamous epithelium with hyperparakeratosis and acanthosis, which had elongated rete ridges and scarce lymphocytic presence in the subepithelial connective tissue (Figure 2(a)). A characteristic finding was the vacuolization of suprabasal keratinocytes and dyskeratotic cells with dense perinuclear eosinophilic condensation, demonstrating cytokeratin filament aggregation (Figures 2(b) and 2(c)).

Histopathological findings were detected as follows:

- Acanthosis and parakeratosis of the squamous epithelium
- The extensive vacuolization of suprabasal keratinocytes
- Keratinocytes with dense perinuclear eosinophilic condensation due to the aggregation of intermediate filaments

The histological changes described above were not pathognomonic. Still, they were typical of the disease and were diagnostic of white sponge nevus when considered along with the clinical features and history, and that is why genetic testing was not performed. Due to the benign and asymptomatic nature of the lesions, no treatment was prescribed. Instead, the nature of the disease was explained to the patient, and guidelines were set to monitor the condition and present for follow-up examinations in the incidence of any new complaints.

Regular follow-up was recommended for the patient twice a year.

## 3. Discussion

The white sponge nevus is an autosomal dominant genodermatosis that often manifests in early childhood and does not show a gender predisposition [2, 8]. In our case, the lesions were long-standing and had not been histologically verified through years.

The autosomal dominant feature of this disease is irregular penetrance, variability, and expressivity within the same family [3–5]. No similar lesions were found among the patient's relatives, and the lesions only affected the oral mucosa. The mucous membranes of the pharynx, esophagus, and genitals were not affected. Keratins include about 30 proteins classified as type 1 or type 2. In white sponge nevus, point mutations involve the keratin four and keratin 13 genes, which encode the mucosa-specific keratin intermediate filament proteins cytokeratin 4 (type 2) and cytokeratin 13 (type 1). These proteins are important for the formation of keratin filaments [4, 5]. Liu et al. compared sporadic and familial cases of white spongiform nevus and concluded that only one in five sporadic cases had keratin mutations [6]. Therefore, the present case was probably sporadic, as no similar familial cases existed.

Lesions are usually seen bilaterally on the oral mucosa and present as symmetrical, thick, white, and diffuse plaques that affect the buccal mucosa and the gingiva of the upper and lower jaws, as observed in our case. Affected mucosa has a whitish, hyperkeratotic, and velvety surface with a soft consistency and varying density [7]. Less common intraoral localizations of white sponge nevus include the tongue, labial mucosa, soft palate, alveolar mucosa, and the floor of the mouth [7, 8]. Histologic findings are characteristic of white sponge nevus, including acanthosis, hyperparakeratosis, and keratinocyte vacuolization with characteristic perinuclear eosinophilic condensation, which is not necessarily pathognomonic [9, 10]. Microscopic examinations reveal hyperkeratotic stratified squamous epithelium covered with a thick parakeratin layer. The surface epithelium is edematous and contains numerous superficial keratohyalin granules (Figure 2). The extensive vacuolization of the suprabasal keratinocytes is also seen.

In addition, characteristic dyskeratotic cells are prominent, demonstrating dense perinuclear eosinophilic cytoplasmic condensation and perinuclear keratinization. This feature is typical of the disease due to the ultrastructural concentration of tonofilaments around the nucleus, which was also found in our case.

The malignant transformation of white sponge nevus is extremely rare. However, Downham and Plezia [11] reported the occurrence of oral squamous cell carcinoma in a case of white sponge nevus during long-term corticosteroid therapy. Various risk factors can trigger malignant transformations (tobacco and alcohol use, HPV, chronic trauma, etc.) [10]. The differential diagnosis of oral white sponge nevus has to consider other conditions and diseases that present as white lesions on the oral mucosa [2, 9, 12].

In establishing the diagnosis, it is essential to adhere to the following clinical steps: (1) determine whether the disease is congenital or acquired; (2) determine whether there is a systemic disease manifesting as lesions in the oral cavity or whether the lesions are only localized to the oral mucosa; (3) determine whether there are single or multiple lesions affecting the oral mucosa; (4) determine whether the lesions can be removed by scraping; and (5) evaluate the lesion age and development dynamics. These steps allow for the assessment of the clinical behavior of the lesions and for other measures to be taken, such as the removal of suspected etiological factors, control examinations after 10–14 days, and additional paraclinical examinations to confirm the diagnosis.

Other congenital diseases that can present with white plaques include leukoedema, follicular keratosis, dyskeratosis congenita, hereditary benign intraepithelial dyskeratosis (Witkop-von Sallmann syndrome), congenital pachyonychia, and Darier disease.

Leukoedema is asymptomatic and clinically presents as veil-like gray-white diffuse plaques. It is more pronounced in smokers and is refractory to scraping, but it does not have the potential for malignant transformation. Therefore, no treatment is required for this condition.

Dyskeratosis congenital (DC), also known as Zinsser–Engman–Cole syndrome, is a bone marrow failure (BMF) syndrome inherited as an X-linked disorder mainly occurring in males and showing fewer clinical manifestations in females. The most critical oral manifestations include bullae formation, followed by erosions that eventually progress to leukoplakia lesions on the buccal mucosa, tongue, and oropharynx, and rapidly progressive periodontal disease. Malignant transformation has been reported in approximately 30% of leukoplakia lesions within 10–30 years [2].

Hereditary benign intraepithelial dyskeratosis (HBID), also called Witkop-von Sallmann syndrome), is a rare autosomal dominant disorder of the conjunctiva and oral mucosa. During childhood, the oral manifestations are seen as thick, corrugated white plaques affecting the buccal and labial mucosa (similar to white sponge nevus), often with added candidiasis. Milder cases show an opalescent appearance that mimics that of leukoedema. When lesions are active, patients may complain of lacrimation, photophobia, and itchy eyes. In addition, dense gelatinous plaques are seen on the cornea and conjunctiva [2].

Acquired white lesions with a differential diagnosis of white sponge nevus that can be removed by scraping are often the result of contact stomatitis, burns with various agents (hypochlorite, oxygen water, etc.), or traumatic lesions (e.g., mucophagia, the pseudomembranous form of candidiasis, and Koplik spots in measles).

The pseudomembranous form of candidiasis manifests as milky white plaques that can be removed by cooling and leaving an erythematous and sometimes bleeding surface. It is often seen in patients with HIV, those using corticosteroid inhalers, and those with diabetes or impaired immune systems [2, 8]. Eliminating predisposing factors and applying systemic or topical antifungal medications are fundamental to treatment if possible.

Other acquired diseases that present with white plaques can be mistaken for white sponge nevus and cannot be removed by scraping, including focal epithelial hyperplasia, oral florid papillomatosis (associated with human papillomavirus), hyperplastic candidiasis, lichen planus, lichenoid changes, leukoplakia, and squamous cell carcinoma. Oral lichen planus (OLP) and lichenoid changes may also be mistaken for white sponge nevus [9, 13–15].

Lichenoid reactions are nonspecific, and their clinical and histopathological features are challenging to differentiate. They often appear as white plaques with reticular/mesh-like structures. Although the etiology of OLP is multifactorial, it is believed that an imbalanced immune system and autoreactive T lymphocytes play significant roles in the development of the disease. Oral lichen planus has different clinical manifestations and can present as papular, reticular, white plaque-like, bullous, erythematous, and ulcerative changes [2, 3, 5, 8]. The white components can be seen as plaques with reticular elements.

OLP may be associated with hepatitis C, dyslipidemia, hypothyroidism, or diabetes [2].

White plaques that cannot be removed by scraping can also be linea alba on the buccal mucosa or, in the case of lichenoid reactions, the result of delayed contact allergic reactions or medication allergies (e.g., reactions to beta-blockers, antibiotics, nonsteroidal anti-inflammatory drugs, and diuretics) [2].

The oral manifestations of systemic lupus, pemphigus, and pemphigoid can also be confused with white sponge nevus when the specifics of the disease are unknown.

The differential diagnosis also includes all leukoplakia lesions [2, 3, 8]. Unlike white sponge nevus, verrucous leukoplakia lesions have clearly defined borders and a high potential for malignancy. Therefore, histopathological examinations are a mandatory element of diagnosis and should be carried out as quickly as possible.

Clinically, oral leukoplakia (OL) presents as irreversible, scraping-resistant, and slightly raised white plaques that may have furrows on their surfaces. These lesions are divided into homogeneous and nonhomogeneous types. Homogeneous lesions have regular, smooth, whitish surfaces and well-defined edges.

The nonhomogeneous form of leukoplakia consists of an erythematous part (erythroleukoplakia or spotted type) or a nodular, erosive, ulcerative, or verrucous exophytic component. In the spotted type, the lesions are mostly white. Verrucous proliferative leukoplakia presents as white plaques with raised, dense, and uneven corrugated surfaces [2, 8].

Oral hairy leukoplakia (OHL) develops in immunosuppressed patients with the Epstein–Barr virus or low CD4+ T lymphocyte levels. OHL indicates AIDS progression in HIV infection but may also occur in other immunodeficiency states. It very rarely affects immunocompetent individuals [2, 8].

It is clinically described as whitish, velvety plaques that cannot be removed by scraping, which affect the borders of the tongue unilaterally or bilaterally. The shape of the plaques varies from light, white vertical stripes to exophytic, striated areas with uneven surfaces.

Oral squamous cell carcinoma (OSCC) accounts for 92–95% of all oral cancers.

It may present as red, white, or combined red-white lesions, with surface textures that are granular, papillary, and verrucous or crusted and fibrinous. There may also be ulcers with thickened bases, a sign of infiltration. Verrucous carcinoma (also known as Ackermann's tumor, smokers' cancer, Buschke–Löwenstein's tumor, oral papillomatosis fluoride, epithelium cuniculatum, and carcinoma cuniculatum) is a rare subtype of oral squamous cell carcinoma, which has different clinical and histopathological characteristics. Verrucous carcinoma presents as asymptomatic, diffuse, well-circumscribed, solid, white plaques with papilliform or verrucous changes on the surfaces, which usually have a slow growth rate, local invasion, and a low tendency to metastasize. A biopsy is mandatory when this disease is suspected [2, 8].

Accurately identifying white sponge nevus via histopathological examination is essential to distinguish it from other more severe and potentially premalignant lesions and other genodermatoses, such as hereditary benign epithelial dyskeratosis, lichen planus, lichenoid drug reactions, pemphigus, pemphigoid, lupus erythematosus, and mucophagia.

While some lesions are benign, others are premalignant or manifestations of systemic diseases. Therefore, timely diagnosis is essential, especially given that the treatment approaches differ for different diseases [3, 15, 16].

No treatment is required for asymptomatic oral white sponge nevus due to its lack of malignant potential and the entirely benign nature of the lesions. However, various therapies have been developed to reduce the plaques of white sponge nevus, including beta-carotene, antibiotics (penicillin, azithromycin, etc.), antihistamines, topical applications of inoic acid, and tetracycline mouthwashes, but without success [10, 17, 18]. The condition is benign, persist through whole life of the patient, and follow-up of the patient includes visits twice a year.

*Home message for clinicians*: WSN is hereditary dyskeratotic hyperplasia of oral mucosa. Biopsy is mandatory to establish the diagnosis. Patient education, reassurance, and regular follow-up are important especially in patient with risk factors.

## 4. Conclusions

In conclusion, the presented case illustrated the difficulties in the differential diagnosis of the wide spectrum of white oral mucosa lesions. As a recommendation, clinicians should be aware of the characteristic clinical presentations of white sponge nevus, which can present in multiple locations within the oral cavity, and its similarity to other white lesions, some of which have the potential for malignancy. The condition can be diagnosed based on clinical and biopsy examinations. The diagnosis of oral white sponge nevus should be established as early as possible to avoid unnecessary treatment and allow for the monitoring of the condition, especially in the presence of other risk factors.
